# Prosigna Assay for Treatment Decisions in Early Breast Cancer: A Decision Impact Study †

**DOI:** 10.3390/jcm13175328

**Published:** 2024-09-09

**Authors:** Ece Esin, Hasan Cagri Yildirim, Berna Oksuzoglu, Fatma Markoc, Sezen Guntekin, Irem Bilgetekin, Fatih Yildiz, Fisun Yukruk, Umut Demirci, Rengul Cetin-Atalay

**Affiliations:** 1 Department of Medical Oncology, Dr. A.Y. Ankara Oncology Education and Research Hospital, University of Health Sciences, Ankara 06540, Turkey; omurbernacakmak.oksuzoglu@sbu.edu.tr (B.O.); irembilgetekin@gmail.com (I.B.); dr.fatihyildiz@hotmail.com (F.Y.); drumutdemirci@gmail.com (U.D.); 2 Department of Medical Oncology, Nigde Education and Research Hospital, Niğde 51100, Turkey; hasan-cagri@windowslive.com; 3 Department of Pathology, Dr. A.Y. Ankara Oncology Education and Research Hospital, University of Health Sciences, Ankara 06540, Turkey; fatmamarkoc@yahoo.com (F.M.); fyukruk@onkoloji.gov.tr (F.Y.); 4 CanSyL, Graduate School of Informatics, Middle East Technical University, Ankara 06800, Turkey; sezenguntekin@hacettepe.edu.tr (S.G.); rengul@metu.edu.tr (R.C.-A.)

**Keywords:** prosigna, PAM50, molecular profiling, ROR

## Abstract

**Introduction:** Therapeutic decisions in early breast cancer are based on clinico-pathological features which are subject to intra- and inter-observer variability. This single-center decision impact study aimed to evaluate the effects of the Prosigna assay on physicians’ adjuvant treatment choices. **Methods:** Between 09/2017 and 02/2018, formalin-fixed tumor samples from 52 newly diagnosed, postmenopausal, hormone receptor-positive, HER2-negative breast cancer (T1–T2; pN0-N1a) patients were analyzed. Pre-test clinical judgements and Prosigna test results were compared. **Results:** The mean age was 59 (42–77). Invasive ductal carcinoma (79.2%), grade 2 (52.8%) and T1c-N0 tumors (43.4%) were represented. There was 40.4% discordance between the pre- and post-test risk of recurrences. No significant change was observed in the clinical intermediate risk category, while there was a net reclassification of low-risk patients into a high Prosigna recurrence risk group. In addition, clinically determined intrinsic subtypes were 34.6% discordant with the Prosigna results, which is largely driven by the reclassification of the luminal A tumors into the Prosigna-assessed luminal B group. Before the Prosigna test, endocrine treatment was the primary choice in 20 patients (39.2%), and chemotherapy was recommended to 31 patients (60.8%). Overall, the Prosigna assay led to a change in treatment choice for one patient. **Conclusions:** Although conventional risk assessment methods are relatively inexpensive with shorter turnaround times, their accuracy and value for risk reduction are suboptimal. According to our results, the Prosigna assay was found to be a relevant tool for the clinical decision making process. Long-term follow-up of these patients will elucidate the potential benefits of using multigene molecular tests as biomarkers for treatment.

## 1. Introduction

Early-stage breast cancer (BC) has a favorable prognosis if treated appropriately in an adjuvant setting. Choosing the best approach for each patient is an ongoing challenge. In everyday practice, clinico-pathological evaluations and immunohistochemical biomarkers help with stratifying patients into recurrence risk groups. 

Established pathological factors include tumor size, involvement of axillary lymph nodes, histologically verified estrogen receptor (ER) and progesterone receptor (PR) status, human epidermal growth factor receptor 2 (HER2) expression and Ki-67 [1]. On the other hand, there is a substantial amount of heterogeneity among BC patients proven with molecular profiling [2]. This heterogeneity may not always concord with clinico-pathological markers. In order to optimize treatment with less toxicity, carefully selecting the right patients for chemotherapy and avoiding systemic treatment when unnecessary is crucial. 

In the era of precision medicine, there is a growing need for biomarkers in BC. Perou’s preliminary DNA microarray analysis and subsequently Sorlie et al.’s investigations on DNA cluster hierarchy resulted in the genomic classification of BC into four intrinsic subtypes: luminal A, luminal B, basal-type and HER2-expressing [2,3]. These intrinsic subtypes differ in clinical outcomes, chemotherapy benefit rate and risks of relapse [3,4] Currently, several validated molecular subtyping assays based on molecular profiling are available. The Prosigna assay is a clinically and analytically validated molecular profiling and risk analyzing tool which utilizes the PAM50 (Prediction Analysis of Microarray) algorithm, which is based on 50 intrinsic subtypes linked by gene cluster analysis [5]. The Prosigna assay measures the expression of the genes included in the PAM algorithm and reports a risk of recurrence score (ROR). In the patient report, the results were presented as a complex response of the PAM50 gene signature algorithm, intrinsic subtype and clinical variables including tumor size and nodal status. The mode of expression of the result, expanded risk determination years (10 years vs. 5–9 years), differentiates the Prosigna test from other first-generation risk stratification tools.

Today, therapeutic decisions in early breast cancer (EBC) are based mainly on clinical and pathological biomarkers which are subject to intra- and inter-observer variability [6]. Meanwhile, guidelines developed by major clinical societies reflect increasing evidence of multiparameter molecular markers and recommend their use if available [7]. This single-center decision impact study aimed to evaluate the effect of Prosigna test results on a physician’s adjuvant treatment choices in ER-PR-positive, HER2-negative early breast cancer. 

## 2. Materials and Methods

**Figure 1:**
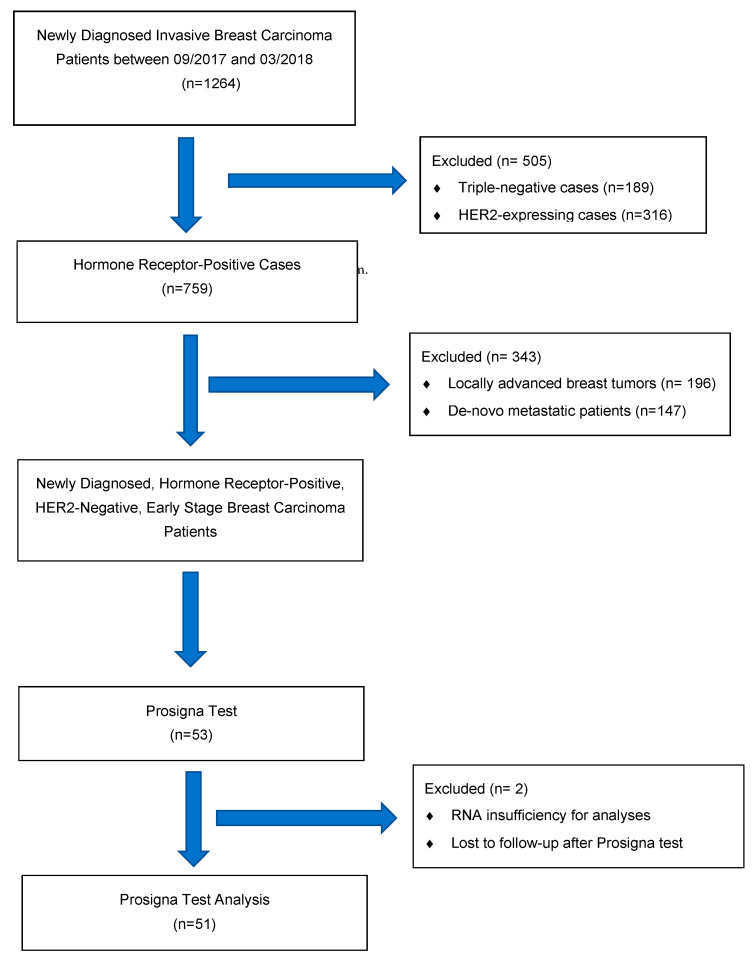
Consort Diagram.

Study Design, Patient Selection, Data Recording and Clinical Risk Assessment: This is a retrospective clinical decision impact study of the Prosigna assay. Newly diagnosed breast cancer patients were recruited from an electronic database from a single comprehensive cancer clinic between September 2017 and February 2018. FFPE tumor samples were sent for Prosigna assay from 53 postmenopausal early-stage (T1-T2; pN0-N1a), hormone receptor-positive, HER2-negative EBC patients. A consort diagram is provided in Figure 1. Demographic data, comorbidities and pathological details were gathered from electronic recordings and patient files. The search for pre-test clinical judgements (risk group and intrinsic subtype) for adjuvant treatment recommendations were collected from the electronic data recording system and recorded in a database. The results of the Prosigna tests (risk of recurrence—ROR score; molecularly defined intrinsic subtype) were collected by a different investigator who was blinded to clinical treatment decisions and were recorded in the study database. 

Of note, risk profiling and intrinsic subtype identifications were carried out by the primary treating physician. The main tool for clinical assessment was local pathological examination and immunohistochemistry (IHC-according to St. Gallen 2013 criteria [8]). Adjuvant treatment proposals were carried out by the primary treating physician in accordance with clinico-pathological factors and medical status. After obtaining the Prosigna result, patients were discussed by the medical oncology tumor board and final decisions were made. 

Pathological Assessment of Tumor Samples: Immunohistochemistry of ER/PR, HER2 and Ki67 were performed locally. ER and PR status were accepted as positive if immunohistochemical staining was above 1%. For the Prosigna assay, formalin-fixed paraffin-embedded surgical specimens were reviewed and prepared in accordance with Prosigna manufacturer’s guide [3] by the local pathologist (investigators FM and FY). 

Prosigna Assay: The Prosigna test was carried out in the local laboratory of the Middle East Technical University Bioinformatics Department. The Prosigna results comprised intrinsic subtypes (luminal A, luminal B, HER2-enriched, basal-like) and ROR risk groups (low-risk, 0–40; intermediate-risk, 41–60; and high-risk, 61–100). These results were reviewed by a blinded investigator (investigator RA) and sent to a data collector (investigator BO). 

Data Analysis: The primary aim of this study was to evaluate the reflection of Prosigna test results on physician’s adjuvant treatment choices in ER-PR-positive, HER2-negative EBC. In addition, the concordance between the clinically defined and the Prosigna-defined risk and intrinsic BC subgroups was investigated. The proportion of patients for whom the physician’s treatment choice changed pre- to post-test (to HT from CT or vice versa) was calculated. Throughout the production of this manuscript, the rules of “REporting recommendations for tumor MARKer prognostic studies (REMARK)” were followed [9]. Data were analyzed using the IBM Statistical Package for Social Sciences (SPSS^®^) v.21 (IBM Inc.; Armonk, NY, USA). The safety margin was accepted as 95% throughout the study. Crosstabs were constructed to summarize bivariate associations between variables, and the kappa coefficient was computed to characterize concordance. 

## 3. Results

Out of 746 newly diagnosed breast cancer patients, 416 ER/PR-positive, HER2-negative EBC patients were identified from a single cancer center (Figure 1). A formalin-fixed paraffin-embedded (FFPE) sample of tumor tissue was sent from 53 patients. For one case, the tumor tissue was RNA-deficient; thus, the Prosigna assay could not be used. One patient was lost to follow-up after the test. Hence, the investigational cohort was defined with 51 cases. 

The median age was 59 (42–77). Patient characteristics are outlined in Table 1. The most common histopathologic presentation was invasive ductal carcinoma (79.2%). Grade 2 (52.8%) and T1c tumors without axillary involvement (43.4%) represented the majority. The Prosigna-assessed luminal A group had a median Ki67 below 15%, whereas the luminal B group of patients had a median Ki67 of 20%. All patients expressed ER in IHC, and three cases were PR negative (5.6%).

The majority of patients were axillary lymph node-negative but in six cases (11.3%) N1a axillary involvement was present. Five of the patients with axillary involvement were classified as high risk after the Prosigna assay and two of them fell into the intermediate-risk category. The percentage of T1 tumors was 84.6% in the Prosigna low-risk group whereas T2 tumors were mostly attributed to high risk (77.8%). Hypertension was the most frequent comorbidity among patients (13 cases, 24.5%), followed by cardiac valvular disease (three cases, 5.6%). Three patients were on mandatory coumadin use due to cardiac valvular disease. 

Clinically multicentric, multifocal disease was present in three of the cases. We performed the Prosigna test on the pathological specimens taken from each of the tumor foci, which displayed differential morphological features in the pathology specimen. The Prosigna scores of these three patients with different tumor foci indicate the same risk scores (low risk, intermediate risk, low risk, respectively). These results indicate that even in the presence of different pathological morphologies, molecular signatures remain the same.

In one of the patients, initial examination of the pathology specimen revealed a hormone receptor-positive grade 2 tumor, which was assessed by the clinician as a luminal B tumor with intermediate risk. The Prosigna test results showed it was Her2-enriched. This discordance contradicted the pathologist and the tumor tissue was analyzed further with SISH, which confirmed HER2 as negative. However, the pathologist concluded that in this patient, the tumor tissue mostly includes carcinoma in situ which may lead to the result of Her2 positivity in the Prosigna test.

Before the Prosigna test, primary treating physicians classified 65.4% of patients as luminal A and 34.6% as luminal B. Pre-test risk groups were marked as 40.4% low risk, 40.4% intermediate risk and 19.2% high risk. 

The Prosigna assay grouped 50% of patients as luminal A, 44.2% as luminal B, 3.8% as basal type and 1.9% as HER2 expressing. The post-test ROR score-based risk groups were as follows: low-risk 25%, intermediate-risk 40.4% and high-risk 34.6%. One patient who was clinically assessed as luminal B was found as HER2-expressing in the Prosigna assay. In order to identify patients’ HER2 status, FISH was applied, which turned out to be negative. Notably, there was no luminal A tumor in the Prosigna ROR high-risk group and only two cases with a luminal B tumor were in the low-risk group (Table 2).

There was a statistically significant correlation between the clinically defined and molecularly assessed (by Prosigna) intrinsic BC subtypes (Table 2, kappa:0.334, *p* = 0.007). Similarly, pre-test and post-test recurrence risk groups were significantly correlated (Table 3, kappa:0.397, *p* = 0.001). Regrouping clinical high-, intermediate-, or low-risk patients according to the Prosigna assay results showed that there was a net upward shift of risk group, especially from the low-risk category (eight upgrades vs. no downgrade, *p* = 0.0001). There was no significant net shift to or from the intermediate category. Additionally, eight clinical luminal A patients were reclassified as molecular luminal B with Prosigna, which is the major responsible factor for the discordance with the intrinsic subtypes (*p* = 0.007). 

Before the Prosigna test, chemotherapy was recommended to 31 patients (60.8%) in the adjuvant treatment. Two of them refused to get chemotherapy and started on endocrine treatment (ET). ET was the physicians’ primary treatment choice in 20 patients (39.2%). 

Patients’ status and treatment selection were re-evaluated by the tumor board after the Prosigna results became available. In one clinically low-risk-attributed patient, the Prosigna assay pointed to a high risk level. For this patient, endocrine therapy was changed to chemotherapy. In the pre-test period, one patient from the high-risk and one patient from the intermediate-risk group were recommended chemotherapy by the primary treating physicians. However, these patients refused to receive chemotherapy. Post-test ROR groups were identified as intermediate risk. These patients were advised to receive chemotherapy again but they refused. Overall, the Prosigna test resulted in a change in adjuvant treatment opinion in 1/51 patients (2%). The change from ET to chemotherapy was for the patient who was assessed as luminal B and high risk by the Prosigna test. The clinical implications of the Prosigna test are presented in Table 4.

Two clinically intermediate-risk patients were on coumadin treatment and there was possible drug interaction of coumadin with the offending cytotoxic agent. ET was initiated for these patients. The Prosigna test results showed that they were luminal A and in the low-risk group. Thus, after the test, the ET decision was more conclusive.

## 4. Discussion

This study aimed to demonstrate the impact of the Prosigna assay on clinical decisions. According to our results, there was 40.4% discordance between the pre-test ROR and post-test ROR. There was no significant net shift to or from the clinical intermediate category, while there was a net upward reclassification of low-risk patients to the high Prosigna ROR group. In addition, clinically determined IHC-based intrinsic subtypes were 34.6% discordant with the Prosigna assay results, which is largely driven by the reclassification of the clinically determined IHC-based luminal A tumors into the Prosigna-assessed luminal B group. 

The average benefit from adjuvant therapy among postmenopausal breast cancer patients varies. A group of cases will be saved from recurrence of disease; on the other hand, some of them will face toxicity in the absence of a net overall improvement in prognosis. An ERpositive, HER2-negative EBC patient has an estimated 10-year risk of ROR. Considering that adjuvant ET will reduce the risk by 30%, one can assume that the addition of adjuvant chemotherapy will result in a remaining 2–3% ROR, which is nearly equal to the observed serious toxicity rate in phase III studies. The absolute risk reduction with hormonotherapy will dictate absolute survival gain; therefore, selection of a patient cohort who will benefit from chemotherapy is crucial. Besides ER and HER2 status, pathological factors like tumor size, axillary lymph node involvement, tumor grade and ki-67 level are used for risk stratification; however, some of these are subject to intra- and inter-observer variability. A biomarker is useful if it identifies the group of patients for whom the absolute benefit of chemotherapy may not exceed 2–3%, which is equal to the risk of serious adverse events. Over the past decade, multiparameter gene profiling has gained importance and has gradually been incorporated into the decision making process. 

Prosigna is a newly developed and validated multigene risk scoring assay [5,10]. It provides two types of information [11]. Firstly, the ROR score, which is a statistically assessed 10-year risk score based on the analysis of 50 genes from tumor tissue [3]. The second result from the Prosigna assay is the genomic intrinsic subtype of tumoral breast tissue. Decision impact studies provide important data of the clinical application and usefulness of multigene assay results on patient management approaches beyond classical risk factors. In particular, whether a patient’s recurrence risk can be further reduced by chemotherapy is the main point of interest. In our study, there was 40.4% discordance between the risk scores and 34.6% discordance between the intrinsic subtype assessments. These results are in parallel with reports of previous decision impact studies [12,13,14,15]. In our study, the major difference was between pre-test luminal A and post-test luminal B intrinsic subtypes, in which 15.4% of patients were re-categorized as luminal B. In addition, there was no significant net shift to or from the clinical intermediate-risk category while there was a net upward re-categorization of low-risk patients into the high Prosigna ROR group. These reclassifications are important for guiding adjuvant treatment decisions. Luminal B subtype and high ROR score mandate adjuvant treatment in most circumstances [16,17]. Intrinsic subtype difference between the IHC-based and molecular-based approaches also highlights that IHC characteristics are not definitive aids for decision making on adjuvant treatment, as major clinical guidelines and the St. Gallen conference consensus suggest [7,18,19].

We also performed the Prosigna test on samples from three patients with three distinct tumor loci and displaying differential morphological features in the pathology examination. The similar Prosigna scores and risk assessment results of these three cancer loci indicate that the RNA expression profiles from different regions of the breast are significant enough to produce risk of recurrence scores without distinct differences. This result indicates that even in the presence of different pathological morphologies, molecular signatures remain the same.

In the present study, the Prosigna assay had a clinical impact in a multitude of ways. First, there was a major change of decision in one patient (1.9%), who was initially identified as low risk in the pre-test period and started endocrine therapy; yet, they were categorized after the Prosigna assay as high ROR. It should be noted that the patient’s primary physician agreed with the Prosigna results. At the end, ET was interrupted for adjuvant chemotherapy. Second, one case in the pre-test intermediate-risk group and one case in the high-risk group were recommended to receive chemotherapy but they refused to take it. A high ROR score on the Prosigna test, on the other hand, did not convince these patients to receive chemotherapy and they continued on ET. In the follow-up of 10 months, no disease recurrence was noted in these patients.

Another clinical implication of the Prosigna test was the increase physician’s confidence in their clinical decision. Two pre-test high-risk cases were on coumadin for valvular heart disease; as such, it was mandatory to continue this drug. However, due to drug interaction, it was impossible to start chemotherapy without halting coumadin. Hence, these patients were started on ET. Post-test results reclassified these patients as low risk. After the test, the ET decision was more conclusive, and primary physicians continued on ET more confidently. The strength of a decision impact study is mainly based on consecutive, clinically assessed patient recruitment from everyday contexts. In this way, patient selection bias could be defeated. The present study also included EBC patients who were clinically assessed and whose adjuvant treatment modality was chosen without a multigene test result. In addition, treatment decisions are based on post-test results and are made by a tumor board; these are two factors which might reduce bias as a result of single-physician assessment. 

This study has limitations. First, the number of patients included in the analysis is low. This limitation is the result of the low number of donated assay kits. At the time that this study was conducted, gene signature-based risk profiling was not readily available for most of the breast cancer patients due to lack of reimbursement. This limitation may create a bias for generalization of the results. Secondly, retrospective design of this study may lead to errors in patient selection and homogenization of the patient group. This is a limitation but, at the same time, this phenomenon created a diversity of breast cancer types due to the limited number of assay kits and the time limitation.

## 5. Conclusions

Although conventional risk assessment methods are relatively inexpensive with shorter turnaround times, their accuracy and value for risk reduction are suboptimal. According to our results, the Prosigna assay was found to be a relevant tool for the clinical decision making process. In cases where there is a discrepancy between the clinical assessment results and the Prosigna assay, tumor boards may guide treatment recommendations. In particular, in a subgroup of patients with potential conflicts in the decision making process, the multigene Prosigna assay may guide physicians more efficiently. Long-term follow-up of these patients will elucidate the potential benefits of using multigene molecular tests as biomarkers for EBC treatment.

## Figures and Tables

**Table 1:** Patient characteristics.

	N = 53	%
Median age (year, range)	59 (37–77)	
T-stage distribution		
T1a	1	1.9
T1b	4	7.5
T1c	23	43.4
2	25	47.2
N-stage distribution		
N0	47	88.7
N1a	6	11.3
TNM Stage		
Stage IA	25	47.2
Stage IB	10	18.9
Stage IIA	15	28.3
Stage IIB	3	5.7
Histologic subgroup		
Invasive Ductal Carcinoma	42	79.2
Invasive Lobular Carcinoma	7	13.2
Mucinous Carcinoma	2	3.8
Mixt carcinoma	2	3.8
Ki67		
<15%	25	47.2
15–29%	16	30.2
>30%	12	22.6
Histologic Grade		
Grade 1	11	20.8
Grade 2	28	52.8
Grade 3	14	26.4

**Table 2:** Correlation of Prosigna and clinically defined intrinsic breast cancer subgroups.

Kappa:0.334 *p* = 0.007		Prosigna Defined Intrinsic Subgroups				
		Luminal A	Luminal B	Basal Type	HER2 Enriched	Total N (%)
Clinically Defined intrinsic Subgroups	Luminal A	22 (64.7)	11 (32.4)	0	1 (2.9)	34
	Luminal B	4 (22.2)	12 (66.7)	2 (11.1)	0	18
Total		26 (50.0)	23 (44.2)	2 (3.8)	1 (1.9)	52 ^a^

HER2: Human epidermal growth factor receptor-2. ^a^ One patient lost to follow-up; investigational cohort included 51 cases. On the other hand, decision criteria and Prosigna test were applied to 52 patients.

**Table 3:** Correlation of Prosigna and clinically defined recurrence risks.

Kappa:0.397 *p* = 0.0001		Prosigna Defined Recurrence Risk (n/%)			
		Low Risk	Intermediate Risk	High Risk	Total
Clinically Defined Recurrence Risk	Low risk	12 (57.9)	8 (36.8)	1 (5.3)	21
	Intermediate risk	1(5)	11 (50)	9 (45)	21
	High risk	0	2 (20)	8 (80)	10
	Total	13 (25)	21 (40.4)	18 (34.6)	52 ^a^ (100)

^a^ One patient lost to follow-up; investigational cohort included 51 cases. On the other hand, decision criteria and Prosigna test were applied to 52 patients.

**Table 4:** Impact of Prosigna results on the final treatment decision.

	Prosigna Low Risk N = 13 (24.5)	Prosigna Intermediate Risk N = 21 (%39.6)	Prosigna High Risk N = 18 (%34)	Total N = 51 ^a^ (%100)
Adjuvan treatment choice before Prosigna Results				
CT + ET	0	12	17	29 (56.9)
ET only	12	7	1	20 (39.2)
CT offered, not accepted by the patient	0	2	0	2 (3.9)
Adjuvan treatment choice after Prosigna results				
CT + ET	0	12	18	30 (58.8)
ET only	12	7	0	19 (37.3)
CT offered, not accepted by the patient	0	2	0	2 (3.9)
Change in adjuvant treatment choice				
From ET to CT	0	0	1	100 (0)
From CT to ET	0	0	0	0 (0)

ET: Endocrine treatment; CT: chemotherapy. ^a^ One patient lost to follow-up; investigational cohort included 51 cases.

## Data Availability

The original contributions presented in the study are included in the article, further inquiries can be directed to the corresponding author/s.
